# Feasibility of computerised positive mental imagery training as a treatment adjunct in in-patient mental health settings: randomised controlled trial

**DOI:** 10.1192/bjo.2021.1042

**Published:** 2021-11-04

**Authors:** Katharina Westermann, Marcella L. Woud, Jan C. Cwik, Christian Graz, Peter W. Nyhuis, Jürgen Margraf, Simon E. Blackwell

**Affiliations:** Mental Health Research and Treatment Center, Faculty of Psychology, Ruhr-University Bochum, Germany; Mental Health Research and Treatment Center, Faculty of Psychology, Ruhr-University Bochum, Germany; Institute of Clinical Psychology and Psychotherapy, University of Cologne, Germany; Psychosomatic Department, Max Grundig Clinic, Germany; St. Marien Hospital Eickel, Germany; Mental Health Research and Treatment Center, Faculty of Psychology, Ruhr-University Bochum, Germany; Mental Health Research and Treatment Center, Faculty of Psychology, Ruhr-University Bochum, Germany

**Keywords:** Positive mental imagery, anhedonia, positive affect, cognitive bias modification, cognitive control training

## Abstract

**Background:**

Positive affect and anhedonia are important but challenging targets for mental health treatments. Previous research indicates the potential of a computerised cognitive training paradigm involving generation of positive mental imagery, termed positive mental imagery training (PMIT), to increase positive affect and reduce anhedonia.

**Aims:**

Our main aim was to investigate the feasibility of PMIT as a positive affect-focused, transdiagnostic adjunct to treatment as usual for patients in in-patient mental health settings.

**Method:**

We ran an open feasibility, randomised controlled trial with three parallel arms: treatment as usual; treatment as usual plus PMIT; and treatment as usual plus an active comparator, cognitive control training. Fifty-seven patients from two different in-patient mental health treatment clinics in Germany were randomised in a 1:1:1 ratio. PMIT and cognitive control training comprised an introductory session followed by eight 15-min training sessions over 2 weeks. Clinical outcomes such as positive affect (primary outcome measure) and anhedonia were assessed at pre- and post-training, and at a further 2-week follow-up.

**Results:**

Adherence was good and attrition was low. The patterns of results for the outcome data were not consistent with a specific effect of PMIT on positive affect, but were more consistent with a specific effect on anhedonia.

**Conclusions:**

The results indicate feasibility and potential promise of a larger efficacy trial investigating PMIT as a treatment adjunct in in-patient mental health settings. Limitations include lack of researcher blinding, small sample size and lack of pre-specified feasibility outcomes. Anhedonia may be a more suitable primary outcome for a future larger trial.

Deficits in positive affect and reduced ability to anticipate or experience enjoyment from activities (i.e. anhedonia) are core components of depression and predict poor prognosis across a range of mental disorders,^1–4^ but do not respond well to current treatments, whether psychological or pharmacological.^5,6^ These outcomes may be improved by augmenting existing evidence-based treatments with a treatment adjunct specifically focused on increasing positive affectivity and reducing anhedonia, and this could have benefits not only in depression, but transdiagnostically across a broad spectrum of mental disorders.^7^ One potential candidate proposed for this purpose is a computerised cognitive training procedure derived from experimental psychopathology research, positive imagery cognitive bias modification (CBM).^8^ Imagery CBM involves repeated resolution of ambiguous training stimuli via generation of positive mental imagery. Initial laboratory-based experimental studies demonstrated increases in state positive affect over single training sessions among healthy participants,^9^ and more recent studies in samples with depression or dysphoria have found increases in positive affect^10^ or reductions in anhedonia^8,10–12^ over longer time frames.

The current feasibility randomised controlled trial (RCT) aimed to provide an initial step in investigating whether positive imagery CBM, here termed positive mental imagery training (PMIT), could provide a useful adjunct to in-patient mental health treatment by increasing positive affect and reducing anhedonia (see the published protocol for extended background and rationale^8^). Novel aspects of this implementation of PMIT requiring feasibility testing included: (a) applicability of the training and measures used to an in-patient sample; (b) a transdiagnostic application focusing on increasing positive affect and reducing anhedonia (rather than depression symptom reduction) across a broad range of diagnoses, and not just in the context of depression; (c) an active comparator, specifically a form of cognitive control training (CCT; see Hoorelbeke and Koster^13^ and Siegle et al^14^ for examples). It was thought that CCT might improve symptoms of depression, but not positive affect or anhedonia, and thus (in a full-scale RCT) allow demonstration of specificity of the effects of PMIT on these outcomes. The study was conducted as a small-scale feasibility RCT, with the main aims of investigating whether a future full-scale RCT designed along the same lines would be feasible and worthwhile to conduct, and informing the design of a potential future trial.

## Method

### Study design

The study was a feasibility RCT with three parallel arms, using a 1:1:1 allocation ratio. Study arms were treatment as usual (TAU), TAU plus PMIT (PMIT group) and TAU plus CCT (CCT group). Outcomes were measured pre- and post-training, and at 2-week follow-up. The authors assert that all procedures contributing to this work comply with the ethical standards of the relevant national and institutional committees on human experimentation and with the Helsinki Declaration of 1975, as revised in 2008. All procedures involving human patients were approved by the Ethics Committee of the Faculty of Psychology, Ruhr-University Bochum (approval number 325). The study was prospectively registered (Clinicaltrials.gov identifier NCT02958228) and the protocol was submitted for publication when recruitment was ongoing.^7^ There were no substantial changes to methods after trial commencement (for full details see Supplementary Material available at https://doi.org/10.1192/bjo.2021.1042). Study materials (with the exception of standardised questionnaires available from the provider), original protocol, computer software for the training interventions, anonymous research data and analysis scripts are available at https://osf.io/gm4fw/.

### Study settings

The study took place in two clinics offering in-patient treatments for mental health in Germany. The initial study site was the Nexus Clinic in Baden-Baden (site 1), Germany, with a first episode of recruitment from November 2016 to January 2017, and a second from October 2017 to December 2017. The St. Marien Hospital Eickel (site 2), Germany, was added as an additional site in March 2017, with recruitment from April 2017 to November 2017. The time windows for recruitment were determined by the availability of a researcher to collect data. Data collection at site 1 was continuous during the recruitment time windows, with one of two different researchers working full time on the project. Data collection at site 2 was intermittent, with one researcher conducting study procedures in addition to other duties.

### Participants and recruitment

Patients admitted to the clinic were informed about the study via personal contact from doctors or psychologists and via announcements in group therapy sessions. Potential participants were provided with an information sheet and, if interested in participating, attended an eligibility assessment where the researcher explained the study in more detail. Written informed consent was then obtained from all participants. A diagnostic interview was conducted and participants completed a demographic questionnaire and a measure of anhedonia, the Dimensional Anhedonia Rating Scale (DARS^15^). Decisions about participant eligibility were made during this assessment, or following discussion with another member of the research team. Inclusion criteria were as follows: aged ≥18 years, sufficient German language skills and receiving treatment in the in-patient clinic during the time frame of the study. The exclusion criterion was existence of a condition or circumstances that could interfere with completion of the study procedures (e.g. severe visual impairment, neurological problem, acute psychosis or substance withdrawal symptoms). If a participant was judged to meet these criteria, a next meeting for the pre-training assessment was arranged.

**Fig. 1:**
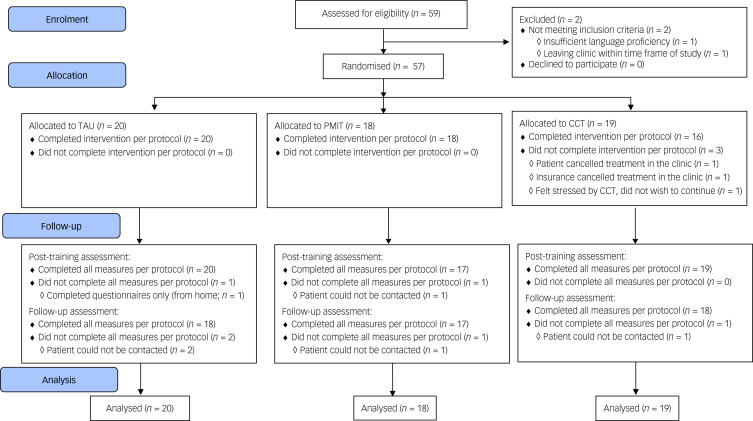
Flow of participants through the trial. CCT, cognitive control training; PMIT, positive mental imagery training; TAU, treatment as usual.

The target sample size was *N* = 90 (i.e. 30 per group) to establish feasibility and provide initial estimates of potential effect sizes; as a feasibility trial, the study was not powered to test or detect between-group differences,^16^ and in this context (a highly heterogeneous sample with all groups receiving active treatment) we would expect between-group effect sizes to be relatively small. However, when the available time frame for recruitment ended only 57 participants had been randomised (see Fig. 1).

### Interventions

#### TAU

TAU included individual and group psychotherapy sessions following primarily cognitive–behavioural therapy principles and other therapeutic activities in the clinic, such as sports, movement, relaxing, music, occupational, creative and art therapy, as well as pharmacotherapy (see Table 1 for details of the main types of medications used). Provision of TAU was independent of the study's research procedures and carried out by clinic staff according to their standard treatment provision, individualised according to each patient's needs. Hence, participants in all treatment groups received TAU as they would normally if they were not taking part in this research.

**Table 1:** Characteristics of participants allocated to TAU, PMIT and CCT

	TAU (*n* = 20)	PMIT (*n* = 18)	CCT (*n* = 19)
Age, years, mean (s.d.)	46.55 (13.32)	43.11 (14.93)	45.53 (11.98)
Gender, female, *n* (%)	9 (45.00%)	9 (50.00%)	10 (52.63%)
Marital status, *n* (%)			
Single	6 (30.00%)	5 (27.78%)	8 (42.11%)
Partnership/married	11 (55.00%)	10 (55.56%)	9 (47.37%)
Divorced	3 (15.00%)	2 (11.11%)	1 (5.26%)
Widowed	0 (0.00%)	1 (5.56%)	1 (5.26%)
Education, *n* (%)			
Doctorate	1 (5.00%)	1 (5.56%)	0 (0.00%)
University degree	9 (45.00%)	7 (38.89%)	8 (42.11%)
Final secondary school examination	5 (25.00%)	2 (11.11%)	3 (15.79%)
Other school level qualification	5 (25.00%)	7 (38.89%)	7 (36.84%)
No educational qualification	0 (0.00%)	1 (5.56%)	1 (5.26%)
Employment status, *n* (%)			
In paid employment	16 (80.00%)	12 (66.67%)	12 (63.16%)
Student/trainee	0 (0.00%)	1 (5.56%)	0 (0.00%)
Self-employed	0 (0.00%)	1 (5.56%)	2 (10.53%)
Not in employment	4 (20.00%)	3 (16.67%)	4 (21.05%)
Pensioner	0 (0.00%)	1 (5.56%)	1 (5.26%)
Household income, *n* (%)^a^			
≥€5100	6 (30.00%)	4 (23.53%)	5 (27.78%)
€3100–€5099	6 (30.00%)	3 (17.65%)	2 (11.11%)
€900–€3099	5 (25.00%)	8 (47.06%)	9 (50.00%)
<€899	3 (15.00%)	2 (11.76%)	2 (11.11%)
Medication, *n* (%)			
Any psychiatric medication	14 (70.00%)	14 (77.78%)	13 (68.42%)
Antidepressant	12 (60.00%)	14 (77.78%)	12 (63.16%)
Anxiolytic	2 (10.00%)	1 (5.56%)	2 (10.53%)
Antipsychotic	4 (20.00%)	5 (27.78%)	5 (26.32%)
Mood stabiliser/anticonvulsant	3 (15.00%)	4 (22.22%)	1 (5.26%)
Other psychoactive (e.g. stimulant)	0 (0.00%)	2 (11.11%)	3 (15.79%)
Psychological diagnoses, *n* (%)			
Depression	15 (75.00%)	10 (55.56%)	10 (52.63%)
Anxiety	4 (20.00%)	9 (50.00%)	4 (21.05%)
Substance misuse/dependence disorder	1 (5.00%)	2 (11.11%)	5 (26.32%)
Others	7 (35.00%)	8 (44.44%)	9 (47.37%)
Duration of clinic stay beforehand, days, mean (s.d.)	17.05 (18.01)	11.50 (11.36)	16.79 (18.87)
Baseline measures, mean (s.d.)			
PANAS-P	45.45 (13.59)	46.94 (13.50)	44.89 (14.93)
DARS	70.06 (20.49)	67.76 (19.62)	75.76 (15.40)
QIDS-SR	12.90 (4.73)	12.44 (5.62)	12.37 (5.75)
GAD-7	10.60 (4.41)	10.22 (5.86)	11.05 (5.54)
PMH	10.40 (5.50)	9.50 (6.62)	9.68 (6.86)
PIT positive	13.00 (4.81)	12.11 (5.43)	13.11 (4.83)
PIT negative	14.60 (5.17)	15.28 (4.71)	15.74 (4.00)
SST	0.31 (0.23)	0.42 (0.35)	0.35 (0.30)

TAU, treatment as usual; PMIT, positive mental imagery training; CCT, cognitive control training; PANAS-P, Positive and Negative Affect Schedule; DARS, Dimensional Anhedonia Rating Scale; QIDS-SR, Quick Inventory of Depressive Symptomatology – Self Report; GAD-7, Generalized Anxiety Disorder-7; PMH, Positive Mental Health scale; PIT, Prospective Imagery Test; SST, Scrambled Sentences Test.

a. PMIT: *n* = 17; CCT: *n* = 18.

#### PMIT

The PMIT was a CBM paradigm adapted from that developed via experimental psychopathology work,^9^ and previous clinical studies in the context of depression.^8,17^ Participants listened to training stimuli, which consisted of descriptions of (mostly) everyday scenarios, and were instructed to imagine themselves in each scenario as it unfolded, feeling actively involved in the scenario and seeing it as if through their own eyes (i.e. ‘field’ perspective^18^). The scenarios were structured so that they started ambiguous (i.e. could end positively or negatively), but always resolved positively (e.g. ‘A friend persuades you to join them for a run. As you both start off, you realise what a *good idea it was to join them and feel full of energy.*’; positive ending in italics). The aim was that via repeated practice in imagining positive resolutions for ambiguous situations, participants would acquire a more adaptive bias to automatically imagine positive resolutions for ambiguous situations in daily life. All scenarios were recorded in a female voice by one of the researchers (K.W.). An initial introductory session included an extended introduction to mental imagery with several examples presented on the computer, followed by four blocks of five training scenarios. Subsequent training sessions (around 15 mins each) consisted of a brief reminder of the main task instructions, followed by five blocks of eight training scenarios. Each training scenario started with a screen displaying the message ‘Close your eyes. Imagine.’ for 1.5 s, after which the screen went blank and the participant heard the scenario through headphones. The scenario was followed by a 2 s pause, followed by a ‘beep’ tone prompting the participant to open their eyes and rate the vividness of their imagery on a scale from 1 (not at all vivid) to 5 (very vivid) by clicking a button on the screen. The program then moved directly to the next scenario. To promote engagement, feedback on the vividness ratings was displayed on the screen between blocks. Participants completed brief measures of state affect at the start and end of each training session. The PMIT was implemented as a Java (Java Development Kit version 1.8.0_144-b01 for Windows, Oracle Corporation, Redwood Shores, CA, USA; see www.oracle.com/java) desktop application. A researcher was present during the training sessions to start the program for the participants and answer any questions if necessary, but once the program started the participants worked through it independently at their own pace.

#### CCT

The CCT was an adaptive Paced Auditory Serial Addition Test, adapted from that implemented in several studies investigating CCT in the context of depression and rumination.^13,14,19^ Participants were presented via headphones with a continuous stream of digits (1–9), and asked to calculate the sum of the last two digits heard by clicking a corresponding button on the screen (1–18). The task difficulty was adaptive to participants’ performance, in that each session started with an interstimulus interval (ISI) of 3000 ms, and this was reduced by 100 ms following four consecutive correct responses or increased by 100 ms following four consecutive incorrect responses. Participants received feedback on their performance via presentation of their ISI and number of consecutive correct/incorrect responses throughout the task. Each session consisted of 400 trials (to result in a session length approximately equal to that for the PMIT), with participants able to pause and take a break at any time. Participants completed brief measures of state affect at the start and end of each training session. The CCT was implemented as a Java desktop application. As with PMIT, a researcher was present during the training sessions to start the program for the participants and answer any questions if necessary, but once the program started the participants worked through it independently at their own pace.

### Measures

Data on the measures’ internal consistency are presented in Supplementary Table 1. For detailed descriptions of measures, see the study protocol.^7^

#### Primary outcome

Our primary outcome was a 21-item positive scale from the extended Positive and Negative Affect Schedule (PANAS-P,^20^ German translation^21^), asking about the experience of different positive emotions over the previous week. The relevant time point for our primary outcome was post-training. Although the study was not intended for hypothesis testing, we pre-specified a primary outcome in line with what we might plan to use as a primary outcome in a future efficacy trial. As experimental studies had shown increases in state positive affect over a single session of PMIT, positive affect over a longer time period seemed a ‘next step’ extension of this work to a clinical sample, and a potential intermediate route to effects on the clinical outcome of anhedonia.

#### Secondary outcomes

##### Clinical outcomes

Clinical outcome measures included: (a) an extended 26-item German version of the DARS,^15,22^ a self-report measure of different facets of anhedonia (desire, motivation, effort and consummatory pleasure) across different hedonic domains (hobbies/ past-times, food/drinks, social activities and sensory experiences); (b) the Positive Mental Health Scale (PMH^23^), a nine-item questionnaire designed to assess positive aspects of mental health; (c) the Quick Inventory of Depressive Symptomatology (QIDS-SR^24,25^) as a brief (16-item) self-report measure of depressive symptoms; and (d) the Generalized Anxiety Disorder-7 (GAD-7)^26,27^ as a brief (seven-item) self-report anxiety questionnaire.

##### Mechanism outcomes

Measures assessing putative mechanisms were administered at pre- and post-training only. The Prospective Imagery Test (PIT^28,29^) is a measure of the vividness with which people can imagine positive or negative events happening in their future. Each assessment included five positive and five negative hypothetical future situations, with a different set at baseline and post-treatment. The Scrambled Sentences Test (SST^30^) was used as a measure of negative interpretation bias. Participants completed a different set of 20 sentences at baseline and post-intervention. A Single-Target Implicit Association Test (STIAT^31^) was used to assess automatic associations between positive/negative affect relevant attributes and targets related to the self.

#### Other measures

A set of computerised self-report questions about mood/cognitions/behaviour over the previous day (e.g. ‘In the last day I felt anxious’), termed ‘monitoring’, were completed at the start of each training session in the PMIT and CCT groups, and on a similar schedule eight times over the 2-week intervention period in the TAU group. Inclusion of ‘monitoring’ in the TAU group was primarily intended to control for certain non-specific aspects of the active interventions (e.g. repeated assessment, meetings with researchers). Clinical information, such as psychiatric diagnosis, was collected via a structured clinical interview, the Diagnostic Interview for Psychiatric Disorders-Open Access (DIPS-OA^32^) or its short version (Mini-DIPS-OA^33^), by researchers who had completed training and certification in its administration. Expectancy was measured via an adapted German^34^ version of Credibility/Expectancy Questionnaire (CEQ^35^). Finally, a feedback questionnaire was developed specifically for the study.

### Procedure

The schedule of measurement and testing was as described previously.^7^ All study assessments were carried out by a member of the research team, who was no longer blind to participant allocation post-randomisation. At the eligibility assessment, demographic information, the structured clinical interview and the pre-training/baseline administration of the DARS were completed, and eligibility was confirmed. If the patient provided written consent to do so, the outcome of the diagnostic assessment was shared with their individual clinical therapist. At the pre-training assessment, participants completed baseline measures (PANAS-P, PMH, QIDS-SR, GAD-7, PIT, SST and STIAT) and were then randomised to one of the three groups (TAU, PMIT or CCT; for details, see the Randomisation section below). Following a brief introduction to their allocated group, participants completed the CEQ. They then completed an initial version of their allocated task (‘monitoring’ questions/PMIT/CCT). Over the subsequent 2-week period, participants in the PMIT or CCT group completed eight sessions of training over a 2-week period, with four sessions planned per week. Participants in the TAU group completed computerised assessment measures (‘monitoring’) on eight occasions, with a schedule similar to that of the training sessions. After completing the final training session, participants completed the post-training assessment (PANAS-P, PMH, DARS, QIDS-SR, GAD-7, PIT, SST and STIAT). Two weeks after the post-training assessment, participants completed the follow-up assessment (PANAS-P, PMH, DARS, QIDS-SR, GAD-7 and the feedback questionnaire). If a participant had been discharged from the clinic by this point, they completed the assessment online or on paper and returned them by post. They then read debriefing information and had the opportunity to ask further questions (either in person or via telephone or email).

### Randomisation

Randomisation was stratified by gender and by clinic, using variable block lengths and implementation via sealed envelopes so that allocation remained unpredictable to trial staff. The randomisation sequence was generated via a statistician, using R^36^ version 3.3.1 for Windows (R Foundation for Statistical Computing, Vienna, Austria; see https://www.R-project.org/). Only the statistician had access to this sequence. The statistician signed and placed the allocations into sequentially numbered opaque envelopes, with the allocation printed in pale ink so that it was not readable through the envelope. The envelope was signed across the seal and tape placed over the signature. Randomisation occurred in the pre-training/baseline assessment after a participant had completed all baseline measures. The administering researcher first photographed the envelope seal, then opened the envelope, and wrote the date, time and participant number on the allocation sheet, and signed and photographed it. These time-stamped photographs provided evidence of appropriate allocation and could be cross-checked against the time stamp for the questionnaire and training data.

### Adverse events monitoring

Before the start of the study, a list of potential adverse events were defined, and researchers monitored their occurrence for each patient: suicidal ideation (score of ≥2 on item 12 of the QIDS); worsening of depression/anxiety symptoms (increase of > a reliable change index on the QIDS or GAD-7 from baseline to follow-up); dropping out of the in-patient treatment against medical advice; terminating the study because of feeling that it was having adverse effects on mental health; self-reported adverse effects of the study; and other events not listed but judged to be adverse events. Adverse events recorded were communicated to the responsible clinicians at the clinic and the broader research team. At the end of the study, the list of adverse events, their severity and relatedness to the study were evaluated by two researchers (K.W. and S.E.B.) and circulated to the broader research team for agreement.

### Statistical analysis

In line with recommendations for pilot/feasibility studies,^16^ the purpose of this study was not hypothesis testing, and thus we did not test the statistical significance of potential differences between treatment arms on the clinical outcome measures. The analyses were carried out with SPSS version 25 for Windows and RStudio version 1.2.1335^37^ for Windows (RStudio, Inc., Boston, MA, USA; see http://www.rstudio.com/) running R version 3.6.1.

Outcome data (primary and secondary outcomes: scales and their subscales) were summarised in the form of both raw means and standardised effect sizes at each time point, with 95% confidence intervals, for both intention-to-treat and per-protocol samples, and both pooled across sites and separately per site. The intention-to-treat sample included all participants randomised to a group. Estimated means, effect sizes and 95% confidence intervals were estimated via fitting of a linear mixed model over the three assessment time points (i.e. a mixed-model repeated measures ANOVA), using the package nlme version 3.1-140.^38^ The per-protocol sample was defined as those participants who completed at least four of the eight training sessions (or measurement sessions in the TAU group), including at least one in the second week of training, and who provided the required outcome data. Means, effect sizes and 95% confidence intervals were therefore computed from the available data. Analyses were also conducted separately by site (see Supplementary Material).

Effect sizes (both within and between) were calculated by dividing the estimated (intention to treat) or observed (per protocol) mean change by the pooled (across all participants) baseline s.d., such that they can be interpreted as a standardised mean difference, with a sample size correction applied (i.e. what Cumming^39^ calls ‘unbiased *d*’, often termed Hedge's *g*). Confidence intervals for the effect sizes were calculated around an uncorrected estimate (see Cumming^39^, p. 305) that used the s.d. of the relevant change score as the denominator (see analysis scripts available online at https://osf.io/gm4fw/ for full details).

## Results

### Recruitment and baseline characteristics

Data collection began in November 2016 and was completed by December 2017. Recruitment stopped after 59 participants had been assessed for eligibility, because of the lack of researcher availability to continue data collection. Of 59 people assessed for eligibility, one person did not meet the inclusion criteria and one person met inclusion criteria but dropped out before randomisation, resulting in 57 patients being randomised (see Fig. 1). Baseline characteristics are shown in Table 1 and Supplementary Table 1. Depression was the most commonly diagnosed disorder in all groups. Other diagnoses included anxiety disorders, substance misuse/dependence disorder and others such as eating disorders.

### Adherence and attrition

Adherence rates were good and comparable across groups (see Supplementary Tables 1 and 2). Attrition was low (see Fig. 1), and all measures were completed per protocol by 54 (94.74%) participants at pre-assessment, 51 (89.47%) participants at post-assessment and 53 (92.98%) participants at the follow-up-assessment. Feedback appeared balanced across the groups, with ratings generally around the middle of the scales, suggesting acceptability of the training/monitoring. However, there were some indications that the participants found CCT more difficult and rated it less positively compared with PMIT or TAU alone (see Supplementary Table 2).

### Main efficacy analyses

#### Intention to treat

Table 2 provides estimated marginal means and effect sizes derived from the mixed-model analysis of clinical outcomes over time and across group. Supplementary Tables 3 and 5 provide estimated marginal means and effect sizes for the mechanisms measures and the subscales of the PANAS-P and the DARS.

**Table 2:** Intention-to-treat clinical outcomes by group

	Baseline (T1), mean [95% CI]	Post-training (T2), mean [95% CI]	Follow-up (T3), mean [95% CI]	Within-group *g* [95% CI]		Between-group *g* (versus PMIT) [95% CI]	
				T1 to T2	T1 to T3	T1 to T2	T1 to T3
PANAS-P							
TAU	45.45 [39.16–51.74]	55.90 [49.39–62.41]	57.08 [48.98–65.18]	0.72 [0.38–1.18]	0.79 [0.28–1.29]	0.02 [−0.37 to 0.39]	−0.01 [−0.39 to 0.38]
PMIT	46.94 [40.32–53.57]	57.64 [50.68–64.61]	58.50 [50.01–66.98]	0.73 [0.37–1.23]	0.78 [0.25–1.28]	−	−
CCT	44.89 [38.44–51.35]	52.26 [45.59–58.94]	50.96 [42.76–59.16]	0.50 [0.12–0.80]	0.41 [−0.08 to 0.76]	0.23 [−0.22 to 0.55]	0.38 [−0.19 to 0.58]
DARS							
TAU	70.06 [61.69–78.43]	71.55 [64.44–78.66]	69.25 [61.89–76.61]	0.08 [−0.31 to 0.46]	−0.04 [−0.39 to 0.30]	0.45 [−0.08 to 0.69]	0.42 [−0.06 to 0.71]
PMIT	67.76 [58.94–76.57]	77.82 [70.18–85.46]	74.93 [67.21–82.65]	0.51 [0.13–1.09]	0.37 [0.02–0.77]	−	−
CCT	75.76 [67.18–84.35]	77.35 [70.06–84.65]	78.72 [71.24–86.19]	0.08 [−0.36 to 0.56]	0.15 [−0.23 to 0.61]	0.44 [−0.08 to 0.69]	0.22 [−0.21 to 0.55]
PMH							
TAU	10.40 [7.56–13.24]	13.45 [10.44–16.46]	14.78 [11.70–17.86]	0.46 [0.21–0.82]	0.66 [0.35–1.13]	0.18 [−0.20 to 0.56]	0.18 [−0.25 to 0.52]
PMIT	9.50 [6.51–12.49]	13.75 [10.55–16.95]	15.05 [11.82–18.28]	0.64 [0.36–0.98]	0.83 [0.46–1.19]	−	−
CCT	9.68 [6.77–12.60]	11.21 [8.12–14.30]	11.88 [8.75–15.02]	0.23 [−0.04 to 0.45]	0.33 [−0.01 to 0.65]	0.42 [0.02–0.79]	0.52 [0.01–0.78]
QIDS-SR							
TAU	12.90 [10.49–15.31]	8.35 [5.78–10.92]	8.85 [6.38–11.32]	0.81 [0.55–1.24]	0.72 [0.37–1.16]	−0.38 [−0.72 to 0.05]	−0.07 [−0.43 to 0.33]
PMIT	12.44 [9.91–14.98]	9.98 [7.24–12.71]	8.77 [6.18–11.37]	0.44 [0.12–0.78]	0.65 [0.28–1.11]	−	−
CCT	12.37 [9.90–14.84]	9.21 [6.58–11.84]	8.99 [6.48–11.50]	0.56 [0.23–0.80]	0.60 [0.23–0.98]	−0.13 [−0.49 to 0.27]	0.05 [−0.35 to 0.42]
GAD-7							
TAU	10.60 [8.23–12.97]	6.45 [4.26–8.64]	7.30 [5.00–9.59]	0.75 [0.57–1.49]	0.60 [0.24–1.18]	−0.36 [−0.67 to 0.10]	−0.15 [−0.48 to 0.28]
PMIT	10.22 [7.73–12.72]	8.02 [5.68–10.36]	7.74 [5.34–10.15]	0.40 [0.05–0.75]	0.45 [0.04–0.91]	−	–
CCT	11.05 [8.62–13.48]	7.89 [5.65–10.14]	8.07 [5.74–10.39]	0.57 [0.23–0.89]	0.54 [0.14–0.98]	−0.18 [−0.52 to 0.24]	−0.09 [−0.44 to 0.32]

Positive within-group effect sizes indicate improvement. Positive between-group effect sizes indicate relative superiority of PMIT versus the relevant condition. T1, time point 1; T2, time point 2; T3, time point 3; PMIT, positive mental imagery training; PANAS-P, Positive and Negative Affect Schedule; TAU, treatment as usual; CCT, cognitive control training; DARS, Dimensional Anhedonia Rating Scale; PMH, Positive Mental Health scale; QIDS-SR, Quick Inventory of Depressive Symptomatology – Self Report; GAD-7, Generalized Anxiety Disorder-7.

The results did not provide any indication of a difference in change in positive affect (PANAS-P, the primary outcome) between the three groups over time, with large within-group effect sizes (*g*) for change within the PMIT and TAU groups, and medium effect sizes within the CCT group. For anhedonia (DARS), there was a medium effect size for improvement within the PMIT group, but negligible change in the other groups (see Table 2). For positive mental health (PMH), there were medium-to-large within-group effect sizes for change within the PMIT and TAU groups, and small effect sizes in the CCT group. For depression (QIDS) and anxiety (GAD-7), the results showed a reduction across all the groups, with medium within-group effect sizes, and the pattern was for greater reduction from pre- to post-training (but not to follow-up) within the TAU and CCT groups compared with the PMIT group.

#### Per protocol

The analysis in the per-protocol sample (*n* = 53) yielded a similar pattern of results (see Supplementary Tables 4 and 6).

### Adverse events

A total of ten adverse events were recorded, of which none were classified as serious adverse events, and only one (‘Self-reported negative effect of the training’) as related to the study: one participant in the CCT group at site 1 reported finding the training very stressful and was sleeping worse as a consequence (constantly thinking about combining numbers). Otherwise, the recorded adverse events occurred in the context of identifiable external stressors or the participant rating the training as beneficial. The most common adverse event (*n* = 5) was the occurrence of suicidal ideation (see Supplementary Material for further details).

## Discussion

This feasibility RCT was a first step in investigating the question of whether a positive imagery CBM cognitive training intervention, PMIT, might be a useful treatment adjunct in in-patient mental health settings, with a specific focus on improving positive affect and anhedonia. The study aimed to provide feasibility and preliminary outcome data to inform potential larger RCTs in the future.

From a feasibility perspective, the data provided by this study are promising: adherence was very good and attrition was low, which is comparable with previous imagery CBM studies.^8^ One adverse event was coded as related to the study (problems sleeping related to completing the active comparator, CCT), but no serious adverse events occurred. The various previously untested aspects of the application of PMIT in this study, such as the in-patient sample, the specific schedule and implementation of the PMIT paradigm used, and the transdiagnostic application (no specific diagnosis required), did not appear problematic. Further, the main outcome measures appeared to be reliable in this sample. No formal feasibility criteria were set, but overall the study is supportive of the feasibility of running such a trial within these in-patient settings.

In terms of effect size estimates, the results do not indicate a pattern of differential change in positive affect, the primary outcome, across the intervention groups. With the caveat of the small sample size, this suggests that the transient increases in state positive affect associated with single sessions of PMIT did not extend to more general increases in positive affect in daily life. However, the results are more consistent with a greater improvement in anhedonia in the PMIT group compared with the other groups. This would be consistent with reductions in anhedonia (as measured by the anhedonia items of the Beck Depression Inventory-II^40^) compared with sham training reported by previous studies.^8,11,12^ In fact, the lack of change in anhedonia within the TAU and CCT groups, particularly given the improvements in depression observed, is striking and consistent with the argument that anhedonia is a particularly treatment-resistant symptom. Positive affect was chosen as a primary outcome in part because it was hypothesised that this may be a direct target of the training, with a subsequent downstream effect on the clinical target of anhedonia. However, with the caveat of the small and heterogeneous sample, the data are more consistent with the hypothesis of a direct effect on anhedonia, and this clinical outcome may be a more appropriate primary outcome measure in future trials.

### Limitations

The study has a number of limitations, some of which reflect the lack external funding for the trial. In relation to the feasibility aspects of the study, a formal feasibility outcome was not pre-specified (e.g. in terms of rates of recruitment, adherence or attrition). Further, although at site 1 recruitment was continuous and we can estimate a recruitment rate (approximately two patients randomised per week), this is more difficult for site 2, as study personnel were only intermittently available for recruitment and testing over the recruitment period. Our feasibility aims were related to the general study procedures rather than these specific sites, and although the two sites provided a contrast in terms of patient characteristics (e.g. income and educational level; see Supplementary Material), they may not be representative of in-patient clinics in other locations; for example, in relation to patient characteristics and therapeutic programmes. Because of practical and resource constraints, all study procedures were carried out at any one time at a particular research site by one researcher. Thus, the researchers were not blind to participant allocation, providing a potential source of bias. For a subsequent fully powered RCT, funding would need to be sought for sufficient personnel to allow blind assessment of outcome measures. Further, the follow-up assessment was only 4 weeks after the baseline. It would be useful for a future RCT to include a longer follow-up period, including diagnostic and clinician-administered assessments, and a more formal process for monitoring of adverse events. In the current feasibility study, TAU was defined as the standard treatment that each participant would normally receive in the clinic, and was directed by the clinic staff independent of the study procedures. However, as the various components of TAU could also affect treatment outcomes, in future trials it would be useful to collect further details what exactly was received by each individual participant during the study (e.g. in addition to pharmacotherapy, number and nature of psychotherapy sessions received, if any) to verify that there were no systematic differences between trial arms. Finally, this study applied the training transdiagnostically, following the suggestion that increasing positive affect and reducing anhedonia may have benefits across many mental disorders.^7^ However, this led to a highly heterogeneous sample in terms of diagnoses and clinical characteristics, which complicates interpretation of outcome measures such as anhedonia, the nature and severity of which will vary across disorders, and increases the variability in the outcome data. Subsequent efficacy trials could consider restricting the sample to a narrower group of patients (e.g. with affective disorders only), and stratifying randomisation by primary diagnosis to ensure these are balanced across treatment arms and to facilitate taking diagnosis into account in the analyses of the outcome data.

### Implications and summary

This study was a first step toward the investigation of PMIT as a treatment adjunct in in-patient mental health settings, with the aim of specifically increasing positive affect and reducing anhedonia. Overall, the study indicates feasibility of such a study, if there was appropriate funding for personnel. Further, this study suggests potential promise for a full-scale RCT, with a pattern of results consistent with the proposal of a specific effect of PMIT in reducing anhedonia, a symptom that is often treatment-resistant.^6,41^ Based on the current study, a future follow-up trial would be recommended to choose the clinical outcome of anhedonia (e.g. as measured by the DARS) as the primary outcome, rather than positive affect. Combined with recent results finding no effect of the active comparator, CCT, on depression symptoms in an in-patient setting,^42^ our results also suggest that an alternative active comparator intervention should potentially be considered; CCT may be more suitable for patients in remission or with residual symptoms.^13,43^ It is also worth noting that the specific PMIT implementation in this study (eight sessions of 15 min each, with standardised scenarios) was a pragmatic ‘best guess’, and as such may not be optimal. For example, more specifically tailored training scenarios, different or tailored schedules of training (e.g. training to a criterion, or provision of optional ‘booster’ sessions^44^), or measures to enhance either learning or its transfer and generalisation (e.g. instructions for active rehearsal of training material between the sessions themselves or scaffolded self-generation of scenario endings^45,46^), may lead to greater efficacy.

The PMIT intervention in this study has been specifically designed for scalability and transportability across research sites; for example, requiring a researcher only to set up the computer (i.e. no specialised input), and programmed with open-source software so that it can be run free of licenses on any suitable computer. This opens up the possibility for any interested researcher to decide independently to apply for funding for, plan and conduct a suitable follow-up trial. If a pattern of results similar to that in this feasibility trial were found in a fully powered and well-conducted RCT, it would suggest a valuable clinical application of PMIT in reducing anhedonia.

## Data Availability

Data, materials, analysis scripts, the original protocol and the preprint are available on the Open Science Framework at https://osf.io/gm4fw/.

## Supplementary material

For supplementary material accompanying this paper visit http://dx.doi.org/10.1192/bjo.2021.1042. click here to view supplementary material
